# Proteomic Identification of Altered Cerebral Proteins in the Complex Regional Pain Syndrome Animal Model

**DOI:** 10.1155/2014/498410

**Published:** 2014-09-16

**Authors:** Francis Sahngun Nahm, Zee-Yong Park, Sang-Soep Nahm, Yong Chul Kim, Pyung Bok Lee

**Affiliations:** ^1^Department of Anesthesiology and Pain Medicine, Seoul National University Bundang Hospital, Seongnam 463707, Republic of Korea; ^2^School of Life Science, Gwangju Institute of Science and Technology, Gwangju 500712, Republic of Korea; ^3^Laboratory of Veterinary Anatomy, College of Veterinary Medicine, Konkuk University, Seoul 143701, Republic of Korea; ^4^Department of Anesthesiology and Pain Medicine, Seoul National University Hospital, Seoul 110744, Republic of Korea

## Abstract

*Background*. Complex regional pain syndrome (CRPS) is a rare but debilitating pain disorder. Although the exact pathophysiology of CRPS is not fully understood, central and peripheral mechanisms might be involved in the development of this disorder. To reveal the central mechanism of CRPS, we conducted a proteomic analysis of rat cerebrum using the chronic postischemia pain (CPIP) model, a novel experimental model of CRPS. *Materials and Methods*. After generating the CPIP animal model, we performed a proteomic analysis of the rat cerebrum using a multidimensional protein identification technology, and screened the proteins differentially expressed between the CPIP and control groups. *Results*. A total of 155 proteins were differentially expressed between the CPIP and control groups: 125 increased and 30 decreased; expressions of proteins related to cell signaling, synaptic plasticity, regulation of cell proliferation, and cytoskeletal formation were increased in the CPIP group. However, proenkephalin A, cereblon, and neuroserpin were decreased in CPIP group. *Conclusion*. Altered expression of cerebral proteins in the CPIP model indicates cerebral involvement in the pathogenesis of CRPS. Further study is required to elucidate the roles of these proteins in the development and maintenance of CRPS.

## 1. Introduction

Complex regional pain syndrome (CRPS) is a rare but serious and painful disorder. Although CRPS can occur following a minor injury, such as a sprain or even soft-tissue blunt trauma, severe intractable pain from CRPS can impair the quality of life. Symptoms and signs of CRPS include sensory changes (allodynia/hyperalgesia), vasomotor changes (temperature asymmetry/skin color change or asymmetry), sudomotor changes (edema/sweating change or asymmetry), and motor or trophic changes [1]. Although the exact pathophysiology of CRPS is not fully understood, several pathological mechanisms, including oxidative stress [2], neurogenic inflammation [3], and alteration in the autonomic nervous system [4, 5], are known to play some roles in its development. Also, psychophysical studies show that CRPS patients have distorted body image and have difficulty in recognizing the size or the position of the affected extremity [6]. The patients get worse when they think about moving the body part, even if they do not move it [7]. Mechanical stimulation of the “virtual (unaffected)” limb reflected in the mirror results in allodynia, which suggests that allodynia and paresthesia can be mediated by the brain [8]. Thus, the distorted body representation of CRPS patients can be treated with mirror therapy [9, 10]. Also, the spreading of symptoms and signs of CRPS from the initial site of presentation to another limb is a well-known phenomenon, which may be due to aberrant central regulation of neurogenic inflammation [11]. These findings highlight the contribution of a cortical pain mechanism in patients with CRPS. Moreover, functional imaging studies provide supporting evidence for the important role of the central nervous system in the pathogenesis of CRPS [12–14], and recent research suggests that changes in cortical structures can contribute to the pathophysiology of CRPS [15].

Thus, the brain seems to play an important role in the development and maintenance of symptoms and signs in patients with CRPS. Some researchers insist that the peripheral changes in CRPS must be understood as a manifestation of changes in the brain [16]. Therefore, we postulated that protein expression would be altered in the CRPS-affected brain. However, there have been no studies on the changes of cerebral protein expression in CRPS. Therefore, to verify our hypothesis, we conducted a proteomic analysis using multidimensional protein identification technology (MudPIT) in a chronic postischemia perfusion (CPIP) rat model, a novel and widely used experimental model of CRPS type 1 [17].

## 2. Materials and Methods

### 2.1. Animals

This study was approved by the Institutional Animal Care and Use Committee of Seoul National University Bundang Hospital (IACUC number 52-2009-033). Male Sprague-Dawley rats weighing 200–250 g had free access to food and water and were housed individually in cages with soft bedding under a 12 h night/day light cycle at a constant temperature of 20–22°C and a humidity level of 55–60%. The animals were acclimatized for at least 1 week prior to the CPIP procedure.

### 2.2. CPIP Model Generation

**Figure 1:**
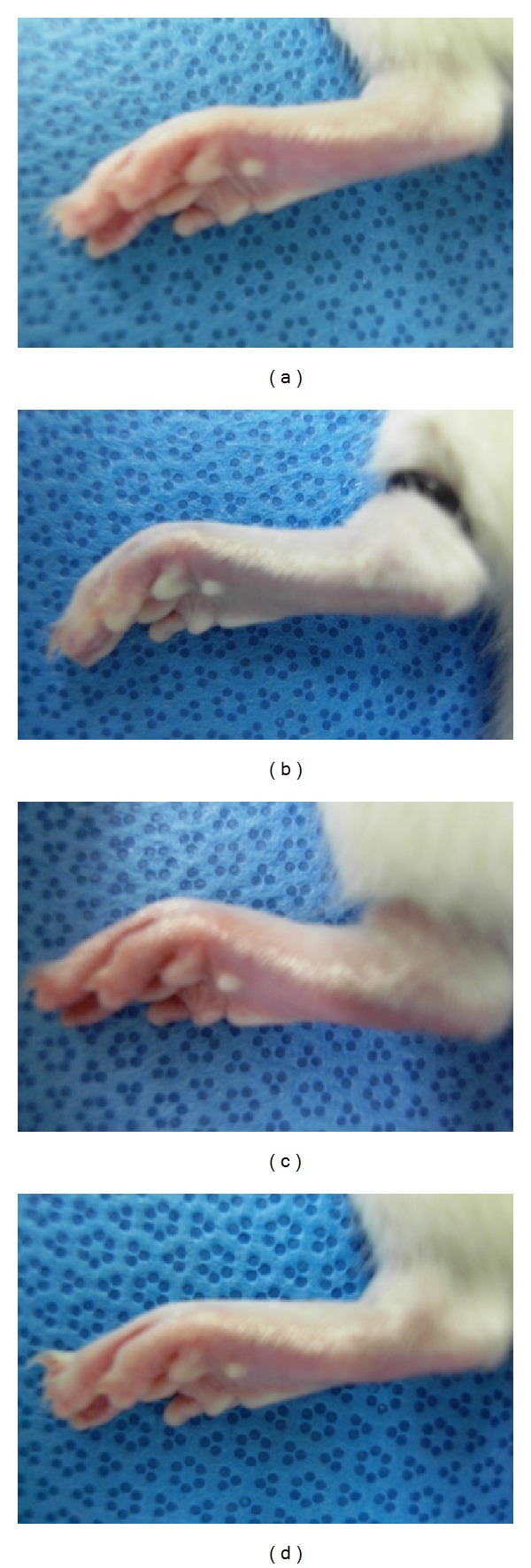
Plantar skin color changes in chronic postischemia pain model rats. (a) Before O-ring application, (b) during O-ring application, (c) 1 hour after reperfusion, and (d) 4 h after reperfusion.

The CPIP animal model was generated according to previous methods [17]. Briefly, after induction of anesthesia with isoflurane, a tight fitting O-ring (O-ring West, Seattle, WA, USA) with a 5.5 mm internal diameter was applied to the left hind limb of each anesthetized rat just proximal to the left ankle joint for 3 h. The O-ring was then removed from the anesthetized rat, allowing reperfusion of the hind limb (Figure 1). The animals in the control group underwent anesthesia similar to the CPIP animals, but the O-ring was not placed around the hind limb.

### 2.3. Behavioral Tests

All behavioral tests were performed during the daylight portion of the regulated circadian cycle between 9 a.m. and 3 p.m. To assess the mechanical threshold, the rats were placed in individual plastic cages with wire mesh bottoms. After 20 min acclimatization, calibrated von Frey filaments (Stoelting Co., Wood Dale, IL, USA) with logarithmically increasing stiffness of 0.41, 0.70, 1.20, 2.00, 3.63, 5.50, 8.50, and 15.10 g were applied to the midplantar surface of the hind paw. The mechanical threshold was assessed using an up-down statistical method [18]. Then, the change in the mechanical threshold (CMT, %) was calculated. The mechanical threshold was examined during the postreperfusion period: 1 h, 4 h, 24 h, 48 h, day 7, and day 21. The CMT was calculated by following equation:

(1) $$\begin{array}{c} \text{CMT}\left(\%\right)=\frac{M_{\text{post}}-M_{\text{pre}}}{M_{\text{pre}}}\times 100. \end{array}$$

We used the findings from the neurobehavioral test on day 21 to classify the animals into groups: rats whose CMT was decreased 50% or more after the CPIP procedure were classified as the successful CPIP (A) group. The mechanical threshold of the animals in the control (C) group was also examined and compared using repeated-measures analysis of variance. All animals were sacrificed 3 weeks after the CPIP procedure for proteomic analysis.

### 2.4. Proteomic Analysis

The difference in cerebral protein expression between Groups A and C was explored using a MudPIT as follows.

#### 2.4.1. Protein Extraction

A total of six animals (three from Groups A and C) were used for the mass spectrometry analysis. On the day 21, right half of each rat cerebrum was grinded using a mortar in liquid nitrogen. The tissue powder was kept at −80°C. The tissue powder was resolubilized in a small volume of 8 M urea, 100 mM Tris-HCl, pH 8.5, and 1 mM dithiothreitol (DTT) for two hours. The homogenates were sonicated and centrifuged at 100,000 ×g for 1 h. Next, 5 mM DTT was added to the homogenate for 30 min at 37°C and alkylated with 25 mM iodoacetamide for 30 min at 37°C in the dark. The samples were then diluted with 2 M urea and with 50 mM Tris-HCl, pH 8.0, and digested at 37°C overnight with sequence grade trypsin (Promega Co., Fitchburg, MA, USA) diluted 1 : 50 in 5 mM CaCl_2_.

#### 2.4.2. MudPIT

Peptides were separated with an Agilent 1100 series high-performance liquid chromatography (HPLC) pump (Agilent technologies, Santa Clara, CA, USA) connected to a linear quadruple ion-trap mass spectrometer (MS, LTQ, Thermo-Finnigan, San Jose, CA, USA) using an in-house-built nanoelectrospray ionization interface. To identify peptides, the ion-trap mass spectrometer was operated in a data-dependent MS/MS mode (*m*/*z* 400–2000), in which a full MS scan was followed by 10 MS/MS scans and the temperature of the heated capillary was 200°C. MS/MS spectra were generated in the positive ion mode at an electrospray voltage of 2.5 kV and normalized collision energy of 35%. An analytical column-fused (100 *μ*m internal diameter) silica capillary microcolumn (Polymicro technologies, Phoenix, AZ, USA) was pulled to a fine tip using a laser puller and packed with 7 cm of 5 *μ*m C18 reverse-phase resin, which was connected to an internal diameter of 250 *μ*m fused-silica trapping column packed with 2 cm of SCX followed by 2 cm of C18 resin. Each 30 *μ*g peptide mixture was manually loaded onto separate columns using a pressure vessel. A seven-step chromatography run was carried out on each sample and three buffers were used (buffer A: 5% ACN/0.1% formic acid, buffer B: 80% ACN/0.1% formic acid, and buffer C: 5% ACN/0.1% formic acid/500 mM ammonium acetate).

#### 2.4.3. Data Searching and Analysis

Acquired MS/MS spectra were searched against an international protein index “rat v. 3.78 FASTA-format decoy database” downloaded from European Bioinformatics Institute (EBI, http://www.ebi.ac.uk/). The SEQUEST algorithm [19] was used to find the best matching sequences from the database with BioWorks 3.3 (Thermo Fisher Scientific Inc., Rockford, IL, USA) for fully tryptic peptides. The mass of the amino acid cysteine was statically modified by +57 Da and the differential modification search was performed for oxidation (+16 Da on Met). Xcorr values were based on tryptic peptides and charge states following 1.8 for singly charged peptides, 2.5 for doubly charged peptides, 3.5 for triply charged peptides, and 0.08 for ΔCn (DTASelect v. 2.0.39). The analysis of protein fold-change was quantified by an overall spectral counting method comparison of label-free methods for quantifying human proteins [20].

## 3. Results

### 3.1. Behavioral Tests

**Figure 2:**
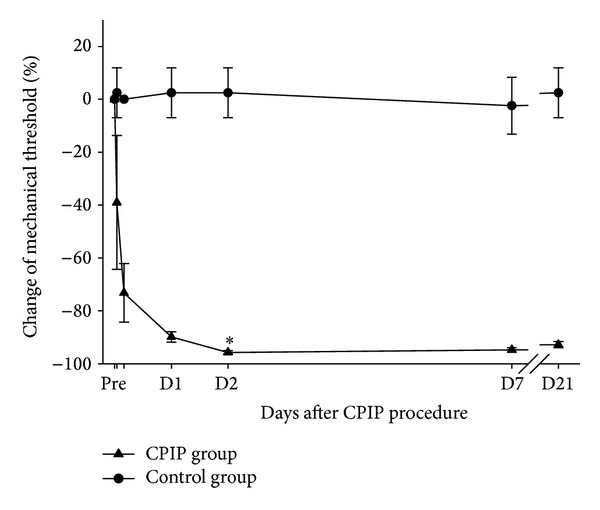
Change in mechanical threshold (%). Group A showed a significant decrease in mechanical threshold compared to group C (*n* = 7 in each group). Asterisk (*) indicates *P* < 0.05 at each time point. Group A: CPIP group and Group C: control group.

A total of 14 animals (*n* = 7 per group) were included in the behavioral test. Before the CPIP procedure, there were no differences in the mechanical threshold between the groups. However, Group A exhibited a significant decrease in the mechanical threshold compared to Group C after the CPIP procedure (*P* < 0.01, Figure 2). The mean differences of CMT (%) in Group A compared to Group C were −41.5, −73.2, −92.3, −98.2, −92.2, and −95.3 after CPIP procedure 1 h, 4 h, day 1, day 2, day 7, and day 21, respectively.

### 3.2. Differential Protein Expression in the Rat Cerebrum

A total of 454 proteins were differentially expressed between Groups A and C under the criterion of *P* value <0.1. Among the 454 proteins, we selected those found in the cerebrum of all study animals in either group and excluded “uncharacterized proteins” and “hypothetical proteins.” Finally, we found 155 differentially expressed proteins between Group A and Group C: 125 increased (Table 6) and 30 decreased (Table 7). Specifically, expression of proteins related to cell signaling (Table 1), synaptic plasticity (Table 2), regulation of cell proliferation (Table 3), and cytoskeletal formation (Table 4) was increased in Group A. Also, expression of a group of protein kinases (calmodulin dependent protein kinase II beta M isoform, casein kinase 2, phosphoenolpyruvate carboxykinase 2, mitogen-activated protein kinase 4, protein kinase C delta, N-terminal kinase like protein, uridine kinase-like 1, serine/threonine protein kinase PLK 1, and phosphoinositide 3 kinase regulatory subunit 4) and calcium-related proteins (inositol 1,4,5-triphosphate receptor type 2, annexin A1, annexin A2, annexin A5, voltage-dependent Ca^2+^ channel gamma-2 subunit, and voltage-dependent Ca^2+^ channel beta-3 subunit, and coiled-coil domain-containing protein 47) was also elevated in Group A. However, several proteins were decreased in group A. Specifically, expression of proteins related to cell signaling (Table 5) and metabolism of fatty acid (peroxisomal 3,2-trans-enoyl Co A isomerase, acetyl-CoA acyltransferase 1b, and acetyl-CoA acetyltransferase 2) were decreased. Also, proenkephalin A, protein cereblon, and neuroserpin were decreased in Group A.

## 4. Discussion

In our study, various proteins were differentially expressed in the cerebrum of CPIP animals. Specifically, expressions of proteins related to cell signaling, synaptic plasticity, regulation of cell proliferation, and cytoskeletal formation were increased in Group A. These findings suggest that both functional and structural changes may occur in the cerebrum of CPIP animals, and altered protein expression can be related to the development of CRPS. This is the first study of cerebral protein expression changes in the CPIP rat model.

We also found that inositol 1,4,5-triphosphate (IP3) receptor type 2 and phosphoinositide 3 kinase (PI3K) regulatory subunit were also increased in Group A. IP3 receptor is intracellular calcium release channel and is regulated by calcium and calmodulin (CaM) [21]. And it is known that PI3K is an important mediator of central sensitization in painful inflammatory condition [22], and many tumorous conditions are related to this enzyme [23, 24]. Based on these findings, cerebral overexpression of IP3 receptor type 2 and PI3K can be related to the sustained pain after rat CPIP model.

Among the 155 proteins expressed differentially, calcium related proteins, including calcium calmodulin kinase II (CaMKII), were increased in group A. Calcium plays a crucial role in many physiological processes, including signal transduction, cell growth, and proliferation. CaMKII is one of the most prominent protein kinases, present in every tissue, but most concentrated in the brain. CaMKII plays various roles, including synthesis and release of neurotransmitter modulation of ion channel activity, synaptic plasticity, learning, and memory [25–28]. Moreover, CaMKII is thought to be important in central sensitization [29–31] and is implicated in central neuropathic pain [31] and long term potentiation (LTP) [32]. LTP is initiated when NMDA receptors allow Ca^2+^ into the postsynaptic neuron, and this Ca^2+^ influx activates CaMKII. LTP in nociceptive spinal pathways shares several features with hyperalgesia, and LTP at synapses constitutes a contemporary cellular model for pain [33, 34]. And it was reported that the overexpression of CaMKII was observed in the dorsal root ganglia of rat model of type 1 diabetes [35], and the inhibition of CaMKII can reverse the chronic inflammatory pain [36]. These findings are consistent with the result of our study. Therefore, overexpression of cerebral CaMKII implicates cerebral involvement in CRPS, and CaMKII can be a target for the prevention and treatment of CRPS.

In addition, we observed that proenkephalin A, cereblon, and neuroserpin decreased in CPIP animals. Proenkephalin is an endogenous opioid hormone which produces the enkephalin peptide. Enkephalin provides a role as inhibiting neurotransmitters in the pathway for pain perception to reduce pain perception. Therefore, decreased proenkephalin A in the cerebrum of CPIP animals seems to reflect the blunted ability to pain modulation and exaggerated response to the pain. For the cereblon, it is known to be related to memory, learning, and intelligence [37], and anomalous cereblon expression can lead to memory and learning deficit [38]. The defect in cereblon gene is associated with mental retardation [39]. Therefore, decreased expression of cereblon in CPIP animals in our study might be related to the deficit in the learning and memory. Neuroserpin, which was known to be related to neurogenesis [40] was decreased in CPIP animals in our study. Neuroserpin plays a role of neuronal protection in pathologic state, and point mutation can cause encephalopathy [41]. Also it has been known that deficiency in neuroserpin exacerbates ischemic brain injury [42]. Therefore, the decreased expression of neuroserpin in CPIP animals in our study might be related to the altered or defected cerebral function.

In our study, we used the CPIP model because CRPS develops after a minor injury without distinguishable nerve lesions. This model is considered a novel animal model of CRPS type 1, in which nerve injury or bone fracture usually does not exist. The previous proteomic studies in neuropathic pain research usually used the nerve ligation model or nerve crush injury model [43, 44]. Since the CPIP model exhibits similar features of human CRPS type 1, our results may have an implication for cerebral involvement in human CRPS. The mechanical threshold was similar at the beginning (day 1) and after 21 days. This is because we took no actions for treatment on the CPIP animals and therefore the initial pain seemed to persist without change. We did not measure the mechanical threshold in the contralateral paws, because it has been already known that the mechanical threshold decreases in the contralateral paws of the CPIP animals, and ipsilateral plantar allodynia is known to be the most characteristic feature of the CPIP animals [17]. The CMT in the ipsilateral paw was used for the criterion of the successful establishment of the animal model.

This study had some limitations. First, the differentially expressed cerebral proteins may not be specific to CPIP animals. These proteins may also change in response to peripheral noxious stimuli. However, CPIP animals exhibit many features of human CRPS type 1, and thus our findings can be extrapolated to human CRPS. Second, because of the complexity of protein interactions in many physiologic pathways in the brain, it is still unclear which is the key protein in the development of CRPS.

Third, we performed proteomic analysis only 21 days after CPIP model generation. However, protein expression related to the development and maintenance of CRPS can differ according to the time course. A proper time course to evaluate a possible correlation between pain behavior development and protein modulation may be useful to discriminate protein changes associated with the early inflammation from that one responsible for possible structural or functional alterations (neural sensitization) occurring at central level. Further investigation of the cerebral mechanism of CRPS is required.

## 5. Conclusion

In conclusion, the cerebral proteome is altered after CPIP injury; many functional and structural changes seem to occur in the cerebrum. These findings support the notion of cerebral involvement in CRPS. Therefore, treatment of CRPS should target not only the periphery, but also the brain.

## Figures and Tables

**Table 1:** Increased cerebral proteins in the chronic post-ischemia pain group; proteins which might be related to cell signaling.

Number	Symbol	Description	*P* value
1	Kctd12	Potassium channel tetramerisation domain containing 12	0.004
2	Ecsit	Evolutionarily conserved signaling intermediate in Toll pathway, mitochondrial	0.004
3	Tns1	Tensin 1	0.004
4	Ccbp2	Chemokine-binding protein 2	0.004
5	Apba1	Amyloid beta A4 precursor protein-binding family A member 1	0.008
6	Tnc	Tenascin C	0.008
7	Rabl2b	RAB, member of RAS oncogene family-like 2B	0.008
8	Epha4	Eph receptor A4	0.035
9	Rab6a	Ras-related protein Rab-6A	0.038
10	Gpr158	G protein-coupled receptor 158	0.042
11	Anxa2	Isoform Short of Annexin A2	0.043
12	Hgs	Isoform 1 of hepatocyte growth factor-regulated tyrosine kinase substrate	0.054
13	Prkcd	Isoform 1 of protein kinase C delta type	0.060
14	Gabarapl2	Gamma-aminobutyric acid receptor-associated protein-like 2	0.065
15	Map2k4	Dual specificity mitogen-activated protein kinase 4	0.066
16	Cacng2	Voltage-dependent calcium channel gamma-2 subunit	0.083
17	Phb2	Prohibitin-2	0.086
18	Camk2b	Calmodulin-dependent protein kinase II beta M isoform	0.086
19	Anxa5	Annexin A5	0.093
20	Scn2a1	Sodium channel Nav1.2	0.095
21	Rab10	Ras-related protein Rab-10	0.097

**Table 2:** Increased cerebral proteins in the chronic postischemia pain group; proteins which might be related to synaptic plasticity.

Number	Symbol	Description	*P* value
1	Itpr2	Inositol 1,4,5-trisphosphate receptor type 2	0.001
2	Kctd12	Potassium channel tetramerisation domain containing 12	0.004
3	Grid2	Glutamate receptor delta-2 subunit	0.004
4	Baiap3	BAI1-associated protein 3-like isoform 2	0.008
5	Atad1	ATPase family, AAA domain containing 1	0.008
6	Pick1	PRKCA-binding protein	0.008
7	Nlgn3	Isoform 1 of Neuroligin-3	0.056
8	Nudc	Nuclear migration protein nudC	0.085
9	Camk2b	Calmodulin-dependent protein kinase II beta M isoform	0.086

**Table 3:** Increased cerebral proteins in the chronic postischemia pain group; proteins which might be related to regulation of cell proliferation.

Number	Symbol	Description	*P* value
1	Pik3r4	Phosphoinositide 3-kinase regulatory subunit 4	0.001
2	Itpr2	Inositol 1,4,5-trisphosphate receptor type 2	0.001
3	Anp32b	Acidic leucine-rich nuclear phosphoprotein 32 family member B	0.002
4	Plk1	Serine/threonine-protein kinase	0.004
5	Drg2	Developmentally regulated GTP binding protein 2-like	0.004
6	Dmwd	Dystrophia myotonica-containing WD repeat motif	0.008
7	Acin1	Apoptotic chromatin condensation inducer 1 protein	0.008
8	Pole2	Polymerase (DNA directed), epsilon 2	0.008
9	Cyld	Ubiquitin carboxyl-terminal hydrolase	0.076
10	Csnk2a2	Casein kinase 2, alpha prime polypeptide	0.084
11	Rab10	Ras-related protein Rab-10	0.097

**Table 4:** Increased cerebral proteins in the chronic postischemia pain group; proteins which might be related to cytoskeletal formation.

Number	Symbol	Description	*P* value
1	Krt4	Keratin, type II cytoskeletal 4	0.008
2	Sntb2	Syntrophin, beta 2	0.008
3	Ckap5	Cytoskeleton associated protein 5	0.038
4	Fermt2	Fermitin family homolog 2	0.063
5	Cotl1	Coactosin-like protein	0.065
6	Rps5	40S ribosomal protein S5	0.072
7	Col1a2	Collagen alpha-2(I) chain	0.079
8	Actr10	Actin-related protein 10 homolog	0.080
9	Etl4	Enhancer trap locus 4-like	0.083
10	Farp1	FERM, RhoGEF (Arhgef), and pleckstrin domain protein 1	0.094

**Table 5:** Decreased cerebral proteins in the chronic postischemia pain group; proteins which might be related to cell signaling.

Number	Symbol	Description	*P* value
1	Vwa1	Von Willebrand factor A domain-containing protein 1	0.002
2	Ppp1r10	Serine/threonine-protein phosphatase 1 regulatory subunit 10	0.003
3	Slc4a8	Isoform 2 of electroneutral sodium bicarbonate exchanger 1	0.003
4	Daam2	Dishevelled associated activator of morphogenesis 2	0.003
5	Trim32	Tripartite motif protein 32	0.007
6	Slc1a1	Excitatory amino acid transporter 3	0.007
7	Spn	Sialophorin	0.007
8	Crbn	Cereblon	0.007
9	Thoc1	Da2-19 THO complex subunit 1	0.007
10	Lmo7	Lim domain only protein 7	0.007
11	Rps27a	Ribosomal protein S27a	0.007
12	Sema4d	Sema domain	0.043
13	Sec3l1	SEC3-like 1	0.047
14	Spna2	Alpha II spectrin	0.065
15	Pde10a	Isoform 3 of cAMP- and cAMP-inhibited cGMP 3′,5′-cyclic phosphodiesterase 10A	0.065
16	Snx2	Sorting nexin 2	0.067
17	Slc25a3	Phosphate carrier protein, mitochondrial	0.068
18	Cox6a1	Cytochrome c oxidase subunit 6A1, mitochondrial	0.090

**Table 6:** Cerebral proteins with increased expression in the chronic postischemia pain (CPIP) group.

Number	Symbol	Description	*P* value
1	Itpr2	Inositol 1,4,5-trisphosphate receptor type 2	0.001
2	Pik3r4	Phosphoinositide 3-kinase regulatory subunit 4	0.001
3	Exoc7	Exocyst complex component 7	0.001
4	Rcor2	REST corepressor 2	0.001
5	Anp32b	Acidic leucine-rich nuclear phosphoprotein 32 family member B	0.002
6	Qrich2	Glutamine rich 2-like	0.002
7	Dnah11	Dynein, axonemal, heavy chain 11	0.002
8	Plk1	Serine/threonine-protein kinase PLK1	0.004
9	Ephx1	Epoxide hydrolase 1	0.004
10	Cacnb3	Voltage-dependent L-type calcium channel subunit beta-3	0.004
11	Anxa1	Annexin A1	0.004
12	Tns1	Tensin 1	0.004
13	Hdac4	Histone deacetylase 4	0.004
14	Osbpl7	Oxysterol binding protein like 7	0.004
15	Ecsit	Evolutionarily conserved signaling intermediate in Toll pathway, mitochondrial	0.004
16	Sorbs3	Sorbin and SH3 domain containing 3, isoform CRA_b	0.004
17	Kctd12	Potassium channel tetramerisation domain containing 12	0.004
18	Ccbp2	Chemokine-binding protein 2	0.004
19	Drg2	Developmentally regulated GTP binding protein 2-like	0.004
20	Grid2	Glutamate receptor delta-2 subunit	0.004
21	Safb	Scaffold attachment factor B1	0.008
22	Dnm3	Isoform 1 of Dynamin-3	0.008
23	Dnajc16	DnaJ homolog subfamily C member 16	0.008
24	Sntb2	Syntrophin, beta 2	0.008
25	Pnpt1	Polyribonucleotide nucleotidyltransferase 1	0.008
26	Eif3g	Eukaryotic translation initiation factor 3 subunit G	0.008
27	Pole2	Polymerase (DNA directed), epsilon 2	0.008
28	Scyl1	N-terminal kinase-like protein	0.008
29	Atad1	ATPase family, AAA domain containing	0.008
30	Krt4	Keratin, type II cytoskeletal 4	0.008
31	Ctsa	Protective protein for beta-galactosidase	0.008
32	Abca1	5 ATP-binding cassette, subfamily A (ABC1), member 15	0.008
33	Dmwd	Dystrophia myotonica-containing WD repeat motif	0.008
34	Baiap3	BAI1-associated protein 3-like isoform 2	0.008
35	Znf512b	Uridine kinase-like 1	0.008
36	Gale	Gale protein	0.008
37	Pick1	PRKCA-binding protein	0.008
38	Acin1	Acin1 protein	0.008
39	Chid1	Chitinase domain containing 1	0.008
40	Pcyox1l	Pcyox1l protein	0.008
41	Rabl2b	RAB, member of RAS oncogene family-like 2B	0.008
42	Serpina3k	Serine protease inhibitor A3K	0.008
43	Glg1	Golgi apparatus protein 1	0.008
44	Tnc	Tenascin C	0.008
45	Lysmd1	LysM and putative peptidoglycan-binding domain-containing protein 1	0.008
46	Apba1	Amyloid beta A4 precursor protein-binding family A member 1	0.008
47	Ckap5	Cytoskeleton associated protein 5	0.038
48	Ndufab1	Acyl carrier protein	0.035
49	Epha4	Eph receptor A4	0.035
50	Kalrn	Isoform 2 of Kalirin	0.035
51	Myh14	Myosin, heavy chain 14	0.035
52	Anxa2	Isoform Short of Annexin A2	0.043
53	Ccdc47	Coiled-coil domain-containing protein 47	0.043
54	Gpr158	G protein-coupled receptor 158	0.042
55	Cugbp1	CUGBP Elav-like family member 1	0.041
56	Hba2	Hemoglobin alpha 2 chain	0.040
57	Acsl3	Isoform long of long-chain-fatty-acid-CoA ligase 3	0.040
58	Rab6a	Ras-related protein Rab-6A	0.038
59	Hbb	Hemoglobin subunit beta-1	0.048
60	Hbb-b1	Zero beta-1 globin	0.044
61	Khsrp	Far upstream element-binding protein 2	0.043
62	Scamp5	Secretory carrier-associated membrane protein 5	0.048
63	Aldh3a2	Fatty aldehyde dehydrogenase	0.049
64	Mesdc2	LDLR chaperone MESD	0.049
65	Rab3d	GTP-binding protein Rab-3D	0.051
66	Vps29	Isoform 2 of vacuolar protein sorting-associated protein 29	0.051
67	Psma3l	Psma3 Proteasome subunit alpha type-3	0.053
68	Hgs	Isoform 1 of hepatocyte growth factor-regulated tyrosine kinase	0.054
69	Nlgn3	Isoform 1 of neuroligin-3	0.056
70	Cygb	Cytoglobin	0.060
71	Pcsk2	Neuroendocrine convertase 2	0.060
72	Prkcd	Isoform 1 of Protein kinase C delta	0.060
73	Fnbp4	Formin binding protein 4	0.062
74	Eif2s3x	Eukaryotic translation initiation factor 2 subunit 3	0.063
75	Fermt2	Fermitin family homolog 2	0.063
76	Vps33a	Vacuolar protein sorting-associated protein 33A	0.063
77	SNX3	Sorting nexin-3	0.063
78	Exoc8	Exocyst complex component 8	0.063
79	Thrap3	Thyroid hormone receptor-associated protein 3	0.063
80	Ndufa1	NADH dehydrogenase (ubiquinone) 1 alpha subcomplex, 1	0.063
81	Gabarapl2	Gamma-aminobutyric acid receptor-associated protein-like 2	0.065
82	Cotl1	Coactosin-like protein	0.065
83	Gad1	Glutamate decarboxylase 1	0.065
84	Ehd1	EH domain-containing protein 1	0.066
85	Map2k4	Mitogen-activated protein kinase 4	0.066
86	Mug1	Murinoglobulin (alpha-1-inhibitor 3)	0.070
87	Pck2	Phosphoenolpyruvate carboxykinase 2	0.072
88	Rps5	40S ribosomal protein S5	0.072
89	Ap2s1	Adaptor protein complex 2 subunit sigma	0.075
90	Tpp1	Tripeptidyl-peptidase 1	0.076
91	Cyld	Ubiquitin carboxyl-terminal hydrolase	0.076
92	Nuc	Nucleolin-like protein	0.079
93	Col1a2	Collagen alpha-2(I) chain	0.079
94	Slc6a17	Orphan sodium- and chloride-dependent neurotransmitter transporter NTT4	0.079
95	Actr10	Actin-related protein 10 homolog	0.080
96	Cacng2	Voltage-dependent calcium channel gamma-2 subunit	0.083
97	Ampd3	AMP deaminase 3	0.083
98	Eif5b-ps1	Eif5b Eukaryotic translation initiation factor 5B	0.083
99	Timm9	Mitochondrial import inner membrane translocase subunit Tim9	0.083
100	Etl4	Enhancer trap locus 4-like	0.083
101	Csnk2a2	Casein kinase 2, alpha prime polypeptide	0.084
102	Cct6a	Chaperonin containing TCP1 subunit 6a	0.084
103	Nudc	Nuclear migration protein nud	0.085
104	Ndufa13	NADH dehydrogenase (ubiquinone) 1 alpha subcomplex 13	0.085
105	Camk2b	Calmodulin-dependent protein kinase II beta M isoform	0.086
106	Clta	Isoform brain of clathrin light chain A	0.086
107	Asah1	Acid ceramidase	0.086
108	Phb2	Prohibitin-2	0.086
109	Sod1	Superoxide dismutase [Cu-Zn]	0.088
110	Ndufs8	NADH dehydrogenase (ubiquinone) 1 alpha subcomplex subunit 8	0.090
111	Slc17a7	Isoform 1 of vesicular glutamate transporter 1	0.091
112	Ugp2	UDP-glucose pyrophosphorylase 2, isoform CRA-b	0.091
113	Rala	Ras-related protein Ral-A	0.091
114	Anxa5	Annexin A5	0.093
115	Hnrph1	Isoform 1 of heterogeneous nuclear ribonucleoprotein H	0.093
116	Stxbp5l	Syntaxin binding protein 5-like	0.093
117	Abcd3	ATP-binding cassette subfamily D member 3	0.094
118	Farp1	FERM, RhoGEF (Arhgef), and pleckstrin domain protein 1	0.094
119	Leng4	Leng4 protein	0.094
120	Scn2a1	Sodium channel Nav1.2	0.095
121	Rab10	Ras-related protein Rab-10	0.097
122	Aldh7a1	Alpha-aminoadipic semialdehyde dehydrogenase	0.097
123	Cltb	Isoform Brain of Clathrin light chain B	0.097
124	Phyhipl	Isoform 1 of phytanoyl-CoA hydroxylase-interacting protein-like	0.098
125	Synpo	Isoform 1 of synaptopodin	0.099

**Table 7:** Cerebral proteins with decreased expression in the chronic postischemia pain (CPIP) group.

Number	Symbol	Description	*P* value
1	Vwa1	Von Willebrand factor A domain-containing protein 1	0.002
2	Ppp1r10	Serine/threonine-protein phosphatase 1 regulatory subunit 10	0.003
3	Poldip2	DNA-directed polymerase delta interacting protein 2	0.003
4	Slc4a8	Isoform 2 of electroneutral sodium bicarbonate exchanger 1	0.003
5	Daam2	Dishevelled associated activator of morphogenesis 2	0.003
6	Cep350	Centrosome-associated protein 350	0.003
7	Tra2b	Transformer-2 protein homolog beta	0.007
8	Epb4.1l1	Isoform L of band 4.1-like protein 1	0.007
9	Trim32	Tripartite motif protein 32	0.007
10	Slc1a1	Excitatory amino acid transporter 3	0.007
11	Spn	Sialophorin	0.007
12	Crbn	Cereblon	0.007
13	Thoc1	Da2-19 THO complex subunit 1	0.007
14	Lmo7	Lim domain only protein 7	0.007
15	Rps27a	Ribosomal protein S27a	0.007
16	Sema4d	Sema domain, immunoglobulin domain (Ig), transmembrane domain	0.043
17	Sec3l1	SEC3-like 1	0.047
18	Ikbkap	Elongator complex protein 1	0.058
19	Peci	Peroxisomal 3,2-trans-enoyl-CoA isomerase	0.058
20	Penk	Proenkephalin-A	0.058
21	Bles03	basophilic leukemia expressed protein	0.058
22	Spna2	Alpha II spectrin	0.065
23	Pde10a	Isoform 3 of cAMP and cAMP-inhibited cGMP 3′,5′-cyclic phosphodiesterase 10A	0.065
24	Snx2	Sorting nexin 2	0.067
25	Slc25a3	Phosphate carrier protein, mitochondrial	0.068
26	Serpini1	Neuroserpin	0.070
27	Acaa1b	Acetyl-CoA acyltransferase 1b	0.077
28	H2afz	Histone H2A.Z	0.079
29	Cox6a1	Cytochrome c oxidase subunit 6A1, mitochondrial	0.090
30	Acat2	Acetyl-CoA acetyltransferase 2	0.094

## References

[1] Harden RN, Bruehl S, Stanton-Hicks M, Wilson PR (2007). Proposed new diagnostic criteria for complex regional pain syndrome. *Pain Medicine*.

[2] Eisenberg E, Shtahl S, Geller R (2008). Serum and salivary oxidative analysis in Complex Regional Pain Syndrome. *Pain*.

[3] Birklein F, Schmelz M (2008). Neuropeptides, neurogenic inflammation and complex regional pain syndrome (CRPS). *Neuroscience Letters*.

[4] Schulze J, Troeger C (2010). Increased sympathetic activity assessed by spectral analysis of heart rate variability in patients with CRPS I. *Handchirurgie, Mikrochirurgie, Plastische Chirurgie*.

[5] Schattschneider J, Binder A, Siebrecht D, Wasner G, Baron R (2006). Complex regional pain syndromes: the influence of cutaneous and deep somatic sympathetic innervation on pain. *Clinical Journal of Pain*.

[6] Moseley GL (2005). Distorted body image in complex regional pain syndrome. *Neurology*.

[7] Moseley GL, Zalucki N, Birklein F, Marinus J, van Hilten JJ, Luomajoki H (2008). Thinking about movement hurts: the effect of motor imagery on pain and swelling in people with chronic arm pain. *Arthritis Care & Research*.

[8] Acerra NE, Moseley GL (2005). Dysynchiria: watching the mirror image of the unaffected limb elicits pain on the affected side. *Neurology*.

[9] Bultitude JH, Rafal RD (2010). Derangement of body representation in complex regional pain syndrome: report of a case treated with mirror and prisms. *Experimental Brain Research*.

[10] McCabe CS, Haigh RC, Ring EFJ, Halligan PW, Wall PD, Blake DR (2003). A controlled pilot study of the utility of mirror visual feedback in the treatment of complex regional pain syndrome (type 1). *Rheumatology*.

[11] Maleki J, LeBel AA, Bennett GJ, Schwartzman RJ (2000). Patterns of spread in complex regional pain syndrome, type I (reflex sympathetic dystrophy). *Pain*.

[12] Schwenkreis P, Maier C, Tegenthoff M (2009). Functional imaging of central nervous system involvement in complex regional pain syndrome. *American Journal of Neuroradiology*.

[13] Freund W, Wunderlich AP, Stuber G (2010). Different activation of opercular and posterior cingulate cortex (pcc) in patients with complex regional pain syndrome (crps i) compared with healthy controls during perception of electrically induced pain: a functional MRI study. *Clinical Journal of Pain*.

[14] Geha PY, Baliki MN, Harden RN, Bauer WR, Parrish TB, Apkarian AV (2008). The brain in chronic CRPS pain: abnormal gray-white matter interactions in emotional and autonomic regions. *Neuron*.

[15] Swart CM, Stins JF, Beek PJ (2009). Cortical changes in complex regional pain syndrome (CRPS). *European Journal of Pain*.

[16] Jänig W, Baron R (2002). Complex regional pain syndrome is a disease of the central nervous system. *Clinical Autonomic Research*.

[17] Coderre TJ, Xanthos DN, Francis L, Bennett GJ (2004). Chronic post-ischemia pain (CPIP): a novel animal model of complex regional pain syndrome-Type I (CRPS-I; reflex sympathetic dystrophy) produced by prolonged hindpaw ischemia and reperfusion in the rat. *Pain*.

[18] Chaplan SR, Bach FW, Pogrel JW, Chung JM, Yaksh TL (1994). Quantitative assessment of tactile allodynia in the rat paw. *Journal of Neuroscience Methods*.

[19] Eng JK, McCormack AL, Yates JR (1994). An approach to correlate tandem mass spectral data of peptides with amino acid sequences in a protein database. *Journal of the American Society for Mass Spectrometry*.

[20] Old WM, Meyer-Arendt K, Aveline-Wolf L (2005). Comparison of label-free methods for quantifying human proteins by shotgun proteomics. *Molecular and Cellular Proteomics*.

[21] Nadif Kasri N, Bultynck G, Sienaert I (2002). The role of calmodulin for inositol 1,4,5-trisphosphate receptor function. *Biochimica et Biophysica Acta*.

[22] Pezet S, Marchand F, D'Mello R (2008). Phosphatidylinositol 3-kinase is a key mediator of central sensitization in painful inflammatory conditions. *Journal of Neuroscience*.

[23] Fung-Leung W-P (2011). Phosphoinositide 3-kinase delta (PI3K*δ*) in leukocyte signaling and function. *Cellular Signalling*.

[24] Lorusso PM, Boerner SA (2010). The role of phosphoinositide 3-kinase in breast cancer: an overview. *Clinical Breast Cancer*.

[25] Yamauchi T (2005). Neuronal Ca^2+^/calmodulin-dependent protein kinase II-discovery, progress in a quarter of a century, and perspective: implication for learning and memory. *Biological and Pharmaceutical Bulletin*.

[26] Solà C, Barrón S, Tusell JM, Serratosa J (2001). The Ca^2+^/calmodulin system in neuronal hyperexcitability. *The International Journal of Biochemistry & Cell Biology*.

[27] Halt AR, Dallapiazza RF, Zhou Y (2012). CaMKII binding to GluN2B is critical during memory consolidation. *EMBO Journal*.

[28] Coultrap SJ, Bayer KU (2012). CaMKII regulation in information processing and storage. *Trends in Neurosciences*.

[29] Fang L, Wu J, Lin Q, Willis WD (2002). Calcium-calmodulin-dependent protein kinase II contributes to spinal cord central sensitization. *The Journal of Neuroscience*.

[30] Dai Y, Wang H, Ogawa A (2005). Ca^2+^/calmodulin-dependent protein kinase II in the spinal cord contributes to neuropathic pain in a rat model of mononeuropathy. *European Journal of Neuroscience*.

[31] Crown ED, Gwak YS, Ye Z (2012). Calcium/calmodulin dependent kinase II contributes to persistent central neuropathic pain following spinal cord injury. *Pain*.

[32] Lisman J, Yasuda R, Raghavachari S (2012). Mechanisms of CaMKII action in long-term potentiation. *Nature Reviews Neuroscience*.

[33] Ruscheweyh R, Wilder-Smith O, Drdla R, Liu X-G, Sandkühler J (2011). Long-term potentiation in spinal nociceptive pathways as a novel target for pain therapy. *Molecular Pain*.

[34] Sandkühler J, Gruber-Schoffnegger D (2012). Hyperalgesia by synaptic long-term potentiation (LTP): an update. *Current Opinion in Pharmacology*.

[35] Ferhatovic L, Banozic A, Kostic S (2013). Expression of calcium/calmodulin-dependent protein kinase II and pain-related behavior in rat models of type 1 and type 2 diabetes. *Anesthesia & Analgesia*.

[36] Luo F, Yang C, Chen Y (2008). Reversal of chronic inflammatory pain by acute inhibition of Ca^2+^/calmodulin-dependent protein kinase II. *Journal of Pharmacology and Experimental Therapeutics*.

[37] Higgins JJ, Tal AL, Sun X (2010). Temporal and spatial mouse brain expression of cereblon, an ionic channel regulator involved in human intelligence. *Journal of Neurogenetics*.

[38] Rajadhyaksha AM, Ra S, Kishinevsky S (2012). Behavioral characterization of cereblon forebrain-specific conditional null mice: a model for human non-syndromic intellectual disability. *Behavioural Brain Research*.

[39] Higgins JJ, Pucilowska J, Lombardi RQ, Rooney JP (2004). A mutation in a novel ATP-dependent Lon protease gene in a kindred with mild mental retardation. *Neurology*.

[40] Yaamada M, Takaehashi K, Ukai W, Hashimoto E, Saito T, Yamada M (2010). Neuroserpin is expressed in early stage of neurogenesis in adult rat hippocampus. *NeuroReport*.

[41] Miranda E, Lomas DA (2006). Neuroserpin: a serpin to think about. *Cellular and Molecular Life Sciences*.

[42] Gelderblom M, Neumann M, Ludewig P (2013). Deficiency in serine protease inhibitor neuroserpin exacerbates ischemic brain injury by increased postischemic inflammation. *PLoS ONE*.

[43] Jiménez CR, Stam FJ, Li KW (2005). Proteomics of the injured rat sciatic nerve reveals protein expression dynamics during regeneration. *Molecular and Cellular Proteomics*.

[44] Alzate O, Hussain S-RA, Goettl VM (2004). Proteomic identification of brainstem cytosolic proteins in a neuropathic pain model. *Molecular Brain Research*.

